# The Knowns and Unknowns in Protein–Metabolite Interactions

**DOI:** 10.3390/ijms24044155

**Published:** 2023-02-19

**Authors:** Ilya Kurbatov, Georgii Dolgalev, Viktoriia Arzumanian, Olga Kiseleva, Ekaterina Poverennaya

**Affiliations:** Institute of Biomedical Chemistry, Moscow 119121, Russia

**Keywords:** protein–metabolite interactions, proteomics, metabolomics, interactomics, macromolecular assembly

## Abstract

Increasing attention has been focused on the study of protein–metabolite interactions (PMI), which play a key role in regulating protein functions and directing an orchestra of cellular processes. The investigation of PMIs is complicated by the fact that many such interactions are extremely short-lived, which requires very high resolution in order to detect them. As in the case of protein–protein interactions, protein–metabolite interactions are still not clearly defined. Existing assays for detecting protein–metabolite interactions have an additional limitation in the form of a limited capacity to identify interacting metabolites. Thus, although recent advances in mass spectrometry allow the routine identification and quantification of thousands of proteins and metabolites today, they still need to be improved to provide a complete inventory of biological molecules, as well as all interactions between them. Multiomic studies aimed at deciphering the implementation of genetic information often end with the analysis of changes in metabolic pathways, as they constitute one of the most informative phenotypic layers. In this approach, the quantity and quality of knowledge about PMIs become vital to establishing the full scope of crosstalk between the proteome and the metabolome in a biological object of interest. In this review, we analyze the current state of investigation into the detection and annotation of protein–metabolite interactions, describe the recent progress in developing associated research methods, and attempt to deconstruct the very term “interaction” to advance the field of interactomics further.

## 1. Introduction

A combination of intermolecular interactions determines the phenotype and functionality of a cell. The total interactome is represented mainly by protein–protein interactions (PPIs) and protein–metabolite interactions (PMIs). The current understanding of the interactions between proteins and small molecules significantly lags behind even the fragmentary knowledge of protein–protein interactions. The current understanding of the interactions between proteins and small molecules significantly lags behind even the fragmentary knowledge of protein–protein interactomes. For example, it is not clear which interactions between proteins to consider relevant [1]. The strength of the interaction can be quantified, but the dissociation constant is known for less than 1% of the protein pairs. In a complex biological matrix, the interaction is determined by a combination of factors of similar behavior of protein molecules under different conditions (guilty by association) [2].

Compared with protein–protein interactions, studying protein–metabolite interaction is more complicated since it depends not only on proteomics techniques but also on methods of metabolomics, which are currently less mature compared to the former [3]. PMIs play a key role in regulating protein functions and directing the orchestra of cellular processes [4,5,6]. The interactions between proteins and metabolites form a highly dynamic adaptive molecular structure that functions harmoniously in healthy cells and tissues and is distorted in pathologies [7].

Knowledge accumulated today is focused mainly on exogenous ligands with promising pharmacological properties [8]. Beyond this area, the complexity of the endogenous protein–metabolite interactome is striking: even in a typical bacterial cell, by the most conservative estimate, functionally significant events may potentially occur between more than one million proteins [9] and 100 million metabolites [10].

Recent advances in mass spectrometry presently allow the routine identification and quantification of thousands of proteins and metabolites, but they still need to be improved to enable a complete inventorization of all biomolecules present in biological objects, e.g., cells. In order to explore a large part of the proteomic iceberg, consisting of low-copy proteins and aberrant proteoforms [11], the proteomic community uses state-of-the-art protocols that are far from unified and often tailored to a particular molecule. Given the unprecedented complexity of the metabolome and the absence of a low-molecular analog of the “proteotypic peptide,” many metabolites are also difficult to distinguish within one chemical class. These limitations naturally complicate the deciphering of biological processes through PMIs.

The complexity of studying the interactome is exacerbated by the fact that existing methods work either in vitro or in vivo but with substantial violation of the natural environment of the cell. The data obtained are contradictory and often do not coincide when analyzed by different methods, which can be caused by both technical reasons and biological ones, for example, in the case of studying different types of cells [1].

The knowledge accumulated today is focused mainly on exogenous ligands that demonstrate potentially valuable properties from a pharmacological point of view. However, the development of high-throughput methods for the exploration of endogenous protein–metabolite interactomes gains speed, offering new opportunities and challenges for the field. The practical demand for information about PMIs contributes to the development of analytical and bioinformatic approaches and advances the omics community in terms of a fundamental understanding of the mechanisms of the interconnection of molecules. In this review, we systematize the current knowledge about PMIs, experimental methods for their detection and annotation, and outline further prospects for protein–metabolite interactomics.

## 2. What Is Protein–Metabolite Interaction?

Molecular interaction is the cornerstone of all cellular processes, many of which are governed by the branched net of contact between proteins and small molecules. During the last decade, the depth of understanding of the molecular machinery of life has advanced dramatically. This progress is fueled by continuously improving technological (mostly mass spectrometric) solutions for exploring the molecular content of biological samples in “omics” mode. Understanding the interactions between the proteomic and metabolomic layers is the second, much more challenging derivative of the task of proteome and metabolome profiling. This complexity is explained primarily by the lack of clear criteria determining what should be considered an “interaction” and what should not.

A pool of covalently bound complexes represents the most investigated area of PMIs, resulting from post-translational modifications or non-enzymatic modifications induced directly by reactive metabolites. However, in interactomics, the focus is generally on non-covalent contacts between proteins and small molecules. These highly dynamic events are particularly exciting to researchers. Such non-covalent binding is characterized by two parameters: specificity and affinity. The degree of specificity distinguishes highly specific (a prerequisite for enzyme-catalyzed reactions) from less specific bindings (e.g., various protein–carbohydrate complexes) [12]. Affinity, in turn, implies binding strength and, in the context of PMI, determines that a high concentration of weakly interacting partners cannot substitute for the effect of a low concentration of the specific partners interacting with high affinity [13]. The difficulty of experimentally “catching” a protein–metabolite pair is explained by the low affinity (generally, mM range and lower) of the interactions [14] and their lightning-fast nature.

**Figure 1:**
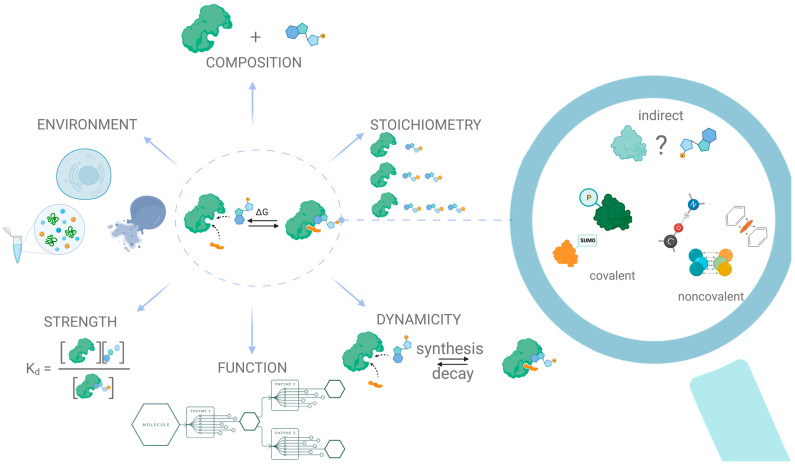
General classification of the type of interaction and challenges in protein–metabolite interactomics.

The “driving force” of PMI is the synergy of energy exchanges between the protein and the small molecule, surrounded by water and buffer ions. Like any spontaneous interaction, binding between a protein and a metabolite occurs if and only if the change in the Gibbs free energy of the system is negative when the system reaches an equilibrium state. The Gibbs energy change is formed from two fundamental components: the enthalpy change and the entropy change, and these components might compensate for each other. Thus, tight binding resulting from multiple noncovalent contacts between metabolites and proteins decreases enthalpy. Such negative changes are often accompanied by a decrease in entropy resulting from the restriction of the mobility of interacting molecules and subsequent changes in binding free energy [15]. In return, the entropy increase aligns with the positive shift in enthalpy due to the energy required to disrupt non-covalent bindings. Several—often underestimated—factors have an impact on this compensation behavior, including the structures of interacting partners, physicochemical characteristics of the solvent, and the balance of forces during binding [16,17,18]. For example, weak, often ignored, interactions can also significantly impact the stabilization of the protein–metabolite complex. Such interactions include π–π stacking, which is typical for molecules with benzene rings and heterocycles [19]. An overview of the current level of understanding of the types of interactions between proteins and metabolites, as well as an outline of trends and challenges in protein–metabolite interactomics, is illustrated in Figure 1.

A complete description of a protein–metabolite complex includes data regarding its composition, formation dynamics, stoichiometry of the interacting partners, strength of the interaction, and the environment that allows the interaction to happen. Thus, a comprehensive description of even a single protein–metabolite interaction is nontrivial and usually requires the utilization of several complementary techniques. Furthermore, there is yet no consensus regarding what to consider an interaction between a protein and a metabolite, since in addition to direct covalent or non-covalent interactions, there exist much weaker and less well-defined interactions, e.g., allosteric interactions, which are significantly harder to identify [20].

Even having sorted out all the components of the Gibbs energy and taking into account all the factors that determine the possibility of interaction between a pair of proteins and metabolites in a truly “panoramic” mode, we can only formulate a very flexible, context-dependent definition of protein–metabolic interaction. By PMI, we mean physical contact between protein and metabolite, implying the formation (and subsequent disturbance) of non-covalent bonds as a result of which the specific physicochemical parameters of each interacting partner change. We detected only the change in these parameters, and from this change inferred the interaction event. However, in the absence of any standardized, physically rigorous definition of PMI, this term has come to mean any positive results from methods that capture a physical association between the protein and the metabolite, meaning that the current use of the term heavily reflects the specifics and biases of the methods utilized. Consequently, in order to advance the field of protein–metabolite interactomics, we need to provide an overview of current state-of-the-art methods for the detection of interactions between proteins and metabolites, highlighting their principles of work, as well as their strengths and weaknesses. Even though the interactomics community has yet to develop a standard definition of PMI, we have many more questions than answers. The entire professional community has nothing left but to eat an elephant of PMIs one bite at a time. Next, we consider the main strategies for eating this elephant and outline prospects for further research in this area.

## 3. Strategies for PMI Studies

The interest in elucidating PMIs has accelerated the development of new methods that rely on the integration of analytical chemistry, synthetic chemistry and systems biology. Traditionally, these methods are divided into three categories: small molecule-to-protein, protein-to-small molecule and panoramic screening. This division separates approaches that aim to identify protein or small molecule targets in panoramic or targeted modes. The major advantage of panoramic approaches over targeted techniques is that discovery methods fuel data-driven research rather than hypothesis-driven research, making them superior for the early stages of biomarker discovery when dealing with complex biological objects. In turn, targeted methods make it possible to focus on the interacting partners of a particular protein or metabolite and to strictly determine the parameters of such an interaction. These and other features of analytical methods and their areas of applicability will be discussed in the following sections.

**Figure 2:**
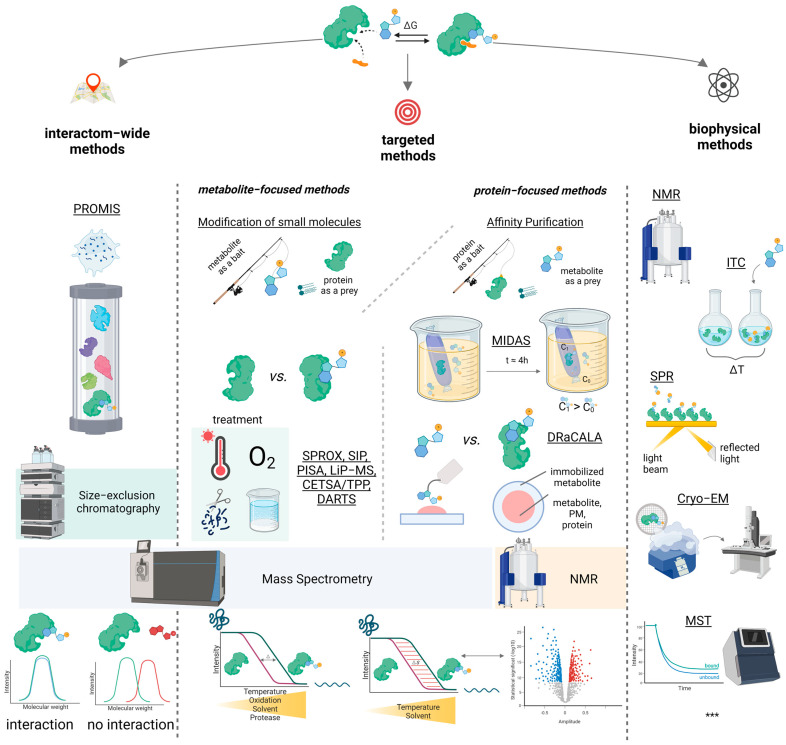
Schematic overview of the classification used in the review and the approaches for studying protein–metabolite interactions discussed in the text. Generally, the approaches are metabolite-focused, protein-focused, interactome-wide, biophysical. A detailed description of these methods (as well as the methods not shown in the illustration, ***) is presented below.

However, the chosen classification system of methods does not take into account such essential characteristics as the need for chemical modification of molecules before their interaction or the possibility of obtaining quantitative information about PMI. In our review, we included methods that deviate from the mainstream classification in the “Modification of small molecules” and “Biophysical approaches” subsections (Figure 2).

### 3.1. Small Molecule to Protein

The variety of PMI methods that start with a particular metabolite is expansive. However, in a broad sense, all of these approaches detect changes in the physicochemical parameters of protein molecules interacting with the metabolite of interest (Figure 2).

#### 3.1.1. Chemoproteomic Profiling

The methods based on the chemical modification of small molecules (in particular, chemoproteomic identification) involve several stages of confirming a non-covalent interaction using individual functional groups [21,22,22]. First, the metabolite is covalently attached to the target protein using so-called “activated reactive” modifications. After that, due to additional sorting groups, the resulting protein–metabolite complexes are removed from the sample for further analysis [23,24]. An illustrative example of the recent application of this technique was provided by Chen et al., who investigated the biological role of acrolein, which is often found in common pollutants [25]. Using an aldehyde-directed aniline-based probe, it was discovered that acrolein interacts with more than 2300 proteins, pointing to the possible biological function of this small molecule.

The principal disadvantage of such methods is the presence of additional groups on the metabolite since they can affect the affinity and selectivity of binding to target proteins. Like affinity-based approaches (see below), chemoproteomic target identification is limited by its ability to synthesize suitable derivatives of the compound of interest. In addition, even when using well-functioning photo-reactive modifications to attach the protein to the bait, there is always the possibility of binding to background proteins, which affects the number of false positive findings [26].

Despite the shortcomings, the method of chemoproteomic identification, with proper planning of the experiment and carefully selected functional groups, minimizes false positive results. This method is a reliable option for orthogonally validating results obtained with other methods and new combinations of metabolites and reactive groups, as well as methods for their activation, are being actively developed [26,27].

#### 3.1.2. Limited Proteolysis

The group of the most common approaches to panoramic exploration of PMIs uses limited proteolysis in combination with mass spectrometry (LiP-MS). The main idea behind this method is to compare the results of the proteolysis of free proteins with those of proteins interacting with metabolites. When interacting with a metabolite, a part of the protein becomes sterically inaccessible to proteases, which ultimately affects the final list of detectable peptides. The experiment includes two stages of proteolysis: first, a non-specific protease is used to digest proteins for a short amount of time in conditions close to native ones for the proteins of interest; then, a more specific protease is used for complete digestion in denaturing conditions. Tryptic peptides quantified by MS allow for the comparison of the structural “fingerprints” of all detected proteins, both free and bound to small molecules [28]. To reduce the influence of endogenous metabolites, which may distort the results, LiP-MS protocols typically use size-exclusion chromatography to separate large protein molecules from smaller metabolites. The purified protein fraction is then incubated with metabolites of interest at a fixed concentration. The more advanced variation of this approach, termed LiP-Quant, involves the incubation of a native cell lysate with small molecules in a concentration gradient. The LiP-Quant approach makes it possible to rank proteins according to the reliability of the detected interaction with the metabolite based on the score obtained using machine learning (LiP-Quant score). The reduction in the proportion of false positive detections in the LiP-Quant method is achieved due to the extensive training set formed based on the results of analyzing many samples in a series of technical repetitions [29,30]. Piazza et al. showed that Lip-Quant can detect PMIs with a wide range of affinities (nM to µM). This method yields reliable results when exploring PMIs in cell lysates and has high potential for application to intact cells, as well as for the identification of metabolite partners of plasma membrane proteins [30]. In pharmacological studies, a similar method, termed drug affinity responsive target stability (DARTS), is widely used to assess changes in the stability of a protein target in response to interactions with a drug molecule [31,32]. In this approach, a small molecule of interest is added to a sample of the protein mixture or cell lysate. The samples (blank one and doped with the small molecule of interest) are digested. The digests are separated by SDS-PAGE and stained to reveal bands of proteins protected from proteolysis by the small molecules, which are submitted to liquid chromatography coupled mass spectrometry (LC-MS) [33,34].

One of the main advantages of LiP-MS methods is their high throughput. For instance, a recent application of LiP-MS to *E. coli* led to the identification of 1678 protein–metabolite interactions (86% of which were detected for the first time), with 2564 proteins identified in total [35]. The same study used LiP-MS to investigate the interactome of the antifungal drug cerulenin in yeast cell extracts and discovered that it interacts only with its designated protein target Fas2, thus confirming the high specificity of both the drug and the method. The main disadvantage of the methods based on limited proteolysis is their non-universality, since the binding of a protein to a metabolite does not obligatory lead to a change in the steric availability for proteolysis.

#### 3.1.3. Rates of Oxidation

The interaction between proteins and metabolites can be detected from changes in the rate of oxidation of methionine residues in the presence of a denaturing agent (e.g., guanidine hydrochloride or urea) [36]. To study protein stability through rates of oxidation (SPROX), samples are treated with increasing concentrations of a denaturing agent in the presence of an oxidizing agent. Protein–metabolite complexes generally show higher stability to denaturation, which is reflected in a shift in the rate of oxidation. This shift is estimated through the increase in the proportion of oxidized methionine residues.

The relatively low frequency of methionine residues in prokaryotic and eukaryotic proteins (ca. 2.5%) limits the applicability of this method [37]]. The method is also not suitable for detecting low-affinity contacts (Kd > 2 mM) [38], including weak regulatory PMIs [8]. Nevertheless, this approach allowed the identification of 139 protein targets of the ATP molecule in yeast cell lysate, thus creating the largest ATP interactome [39]. The primary source of false positive results (ca. 2%) is random errors associated with the quantification of isotopically labeled (e.g., iTRAQ) peptides [40]; however, an addition of technical repetition lowers the proportion of false finds.

#### 3.1.4. Thermal Shift

Cellular thermal shift assay (CETSA), combined with mass spectrometry (also known as thermal proteome profiling (TPP) [41]), allows the study of PMIs in panoramic mode under conditions close to native ones, including directly in intact cells [42,43]. This method is based on the idea that binding to a metabolite can change the thermal stability of a protein. Protein–metabolite complexes may resist elevated temperatures and remain stable, while free proteins are denatured. To study changes in the thermal stability of proteins using CETSA/TPP, protein extracts, intact cells or tissue samples are heated in the presence or absence of small molecules. Denatured proteins are removed by centrifugation and soluble proteins are MS-quantified to plot protein content vs. temperature. Comparing the melting temperatures of a protein interacting with small molecules and a free protein in the blank sample, one can register changes in the thermal stability of the protein and, consequently, suppose PMI [42,44,45]. As an example of MS-CETSA application, researchers utilized it to identify protein targets of antimalarial drugs quinine and mefloquine in *Plasmodium falciparum*, which causes malaria in humans [46].

Thermal shift-based methods are being actively improved. For instance, Shaw et al. used microplates to develop an approach with significantly improved throughput termed HT-CETSA [47]. This method was recently applied to the screening of 896 B-Raf kinase inhibitors [48]. Thermal shift-based methods have an important limitation in that proteins with a melting temperature outside of the range of 40–75 °C (which is typical for most proteins) require more steps of temperature iteration for confident results, thus lowering the robustness of the method [43]. Moreover, it should be taken into account that not all interacting metabolites affect protein stability; therefore, CETSA, like other methods based on assessing the stability of molecular complexes, is prone to false negative results [49]. Despite the above-mentioned disadvantages, the possibility of using CETSA in intact cells is an indisputable advantage of this method, since lysis can disrupt true interactions and give rise to nonspecific ones.

Another method that leverages thermal profiling is Proteome Integral Solubility Alteration (PISA), which does not use temperature-stability graphs. Similar to CETSA/TPP, aliquots of both experimental (with added metabolite of interest) and control samples are incubated at different temperatures. In the next step, aliquots are pooled back together and the protein abundance change is measured between such combined experimental and control samples, rather than between each aliquot pair [50]. The protein abundance change obtained this way is linearly correlated with the difference in melting temperatures in CETSA/TPP methods, given that the protein melting temperature is in the range of 37–67 °C [37]. Accordingly, PISA has an obvious advantage over CETSA/TPP in that it needs significantly fewer LC/MS runs for the same amount of samples, and the freed resources can be used to increase the number of technical and biological replicates or even test more metabolites [51]. With the help of the PISA technique, the antimicrobial drug pyrimethamine was shown to interact with dihydrofolate reductase (DHFR), and this interaction was responsible for the inhibition of STAT3 transcriptional activity induced by pyrimethamine [52].

#### 3.1.5. Resistance to Precipitation

Another MS-based method used for PMI discovery is solvent-induced protein precipitation (SIP) [53]. SIP is based on the idea that metabolite-bound proteins are more resistant to solvent-induced precipitation, as a protein–metabolite complex has a lower energy state and thus requires more energy to unfold it. In this method, a mixture of solvents is added to the experimental (metabolite-treated) and control samples to induce protein precipitation. Precipitated proteins are removed with centrifugation, and supernatant fractions are then quantitatively analyzed with mass spectrometry, revealing differences in protein abundances between experimental and control samples. Solvent proteome profiling was utilized to confirm the intracellular targets of several clinically relevant compounds—SCIO-469(inhibitor of p38 MAP kinases), Alisertib (inhibitor of Aurora kinase A) and MK2206 (inhibitor of AKT) [54]. The workflow is very similar to the TPP method, but these two techniques use different physical principles to induce protein precipitation. Consequently, these methods can complement each other well because if the temperature stability of a protein does not change significantly upon interaction with a metabolite, its solvent-induced precipitation resistance might change and vice versa [53]. In addition, chemical denaturation is more rigorous than thermal denaturation [55].

The advantages of the SIP method include ease of implementation and the ability to determine the affinity of a metabolite (or a drug) for a protein using dose-dependent analysis. As with thermal shift-based methods, the main disadvantage of SIP is that it is not universal [55].

#### 3.1.6. Affinity Chromatography (AC)/Affinity Precipitation (AP)

One of the first methods developed for detecting intermolecular interactions in close-to-native conditions is affinity chromatography (AC), also known as affinity purification (AP). This method features cross-linking of small molecules of interest with the matrix, such as agarose, which are then packed in a chromatography column. Samples, e.g., cell lysates, are then passed through the column, and proteins that interact with the metabolite of interest stay in the column, while the other proteins are eluted. Affinity-based methods have various modifications and are often used together with protein labeling approaches for increased protein target detection rate [56,57]. In one of the recent applications of AC/AP, it was used to identify the interaction between 2′,3′-cyclic adenosine monophosphate (2′,3′-cAMP) and RNA-binding protein Rbp47b, which was later shown to promote stress granule formation in *A. thaliana* seedlings treated with a combination of light and heat stress [58].

The immobilization of small molecules can affect both the affinity and specificity of the interaction, so AC/AP is limited to studying only a handful of stable interactions. Most of the weak noncovalent interactions are destroyed during affinity purification [59]. Another problem with this approach is a relatively high rate of false positive results. Negative controls (e.g., blank beads) or iterative washing of the reaction mixture with chemically related compounds and/or increasing the concentration of the metabolite help to increase the specificity of the method [60]. Orthogonal technologies, such as SPROX [61], CETSA/TPP [62] or LiP-MS [35] are commonly used to validate findings from AC/AP.

### 3.2. Protein to Small Molecule

In many cases, the task of identifying a certain PMI originates from the interest in a particular protein being a potential drug target or therapeutic agent. Along with methods for identifying the protein receptors of a preselected small molecule, several approaches have been developed to study small molecules interacting with a target protein.

Unfortunately, most of these methods are limited by the availability of purified proteins and the use of incomplete small molecule libraries. Recent examples of protein-to-small molecule strategies include nuclear magnetic resonance (NMR) with ligand detection [63], differential radial capillary action of ligand assay (DRaCALA [64]), and mass spectrometry integrated with equilibrium dialysis for the discovery of allostery systematically (MIDAS [65]).

Ligand-detected nuclear magnetic resonance (NMR) spectroscopy is a method utilized to analyze changes in metabolite properties when they interact with the protein of interest. This method was used to study PMIs between several proteins and a couple dozen metabolites, and several new interactions were detected in addition to the ones already known [66]. However, until the exact rate of false positive results is established for this method, its large-scale use will remain restricted. Identifying protein-binding metabolites is only partially objective: systematic bias occurs because candidate metabolites must be preselected; otherwise, only certain chemical classes of compounds can be detected [49].

Another approach to studying protein–metabolite interactions from the protein point of view is the differential radial capillary action of ligand assay (DRaCALA). A mixture of proteins with an isotopically labeled metabolite standard is applied on a nitrocellulose membrane. Nitrocellulose membranes are able to retain proteins due to hydrophobic interactions, while free metabolites can move through nitrocellulose under the action of capillary forces. This method is based on the difference between the mobility of free and protein-bound metabolites [67]. Knowing the exact concentrations of the protein and the metabolite and the ratio of the signal from the isotope inside the protein-containing zone to the signal outside it, it is possible to calculate the bound fraction of the metabolite. Using DRaCALA, 9 out of 20 known and 12 new (p)ppGpp target proteins were identified in the model organism *E. coli* K-12, which demonstrates the analytical capabilities of this method [68]. Despite the promise of this approach, it is not very popular, probably due to the low availability of isotopically labeled metabolites and the existence of more efficient methods (e.g., NMR).

One more not so widely used “protein to metabolite” approach is the integration of mass spectrometry with equilibrium dialysis (mass spectrometry integrated with equilibrium dialysis for the discovery of allostery systematically, MIDAS [65]). The first step of the method is the equilibrium dialysis of a purified protein against a mixture of biologically significant small molecules, i.e., the isolation of a purified protein from a sample containing a complex mixture of metabolites by passing it through a partially permeable membrane. The metabolites (potential protein partners) reach concentration equilibrium on both sides of the membrane. In this case, the total concentration of the metabolite (including free and bound to the protein) is increased on the side that contains the protein interacting with the metabolite. Finally, the relative concentration of each metabolite in both the protein-containing chambers and in the empty control chambers is quantified using mass spectrometry coupled with liquid or gas chromatography. Despite its simplicity, MIDAS did not find wide application, presumably due to the large amount of purified protein needed.

The tandem affinity purification (TAP) allows the metabolomic identification of interacting small molecules under close to native conditions to be carried out. It is an adaptation of the AP protocol, which is traditionally used to analyze protein–protein interactions. A protein of interest is epitopically labeled and produced in cells. The protein and metabolite complexes are then immunoprecipitated from the native cell lysate with the help of immobilized antibodies designed to recognize the epitope. Finally, partner proteins and metabolites are analyzed using a mass spectrometric platform. The main disadvantage of TAP is its tendency to produce false positive results due to the presence of an epitope tag and non-specific binding to the matrix. Non-specific interactions are usually excluded using several negative controls, such as a vector control with an empty epitope tag, other proteins, several purification steps, and/or subcellular localization filters. Due to the similarity of this method to approaches for studying PPIs, TAP provides an opportunity to study PMI and PPI in parallel under close to physiological conditions, which distinguishes this method from the other approaches described above [69]. In one of the recent demonstrations of this approach, more than 1200 proteins and 30 metabolites were identified as interacting partners of nucleoside diphosphate kinases (NDPK) in *Arabidopsis thaliana*, which led to the identification of a new mode of regulation of these enzymes [70].

### 3.3. Non-Targeted Methods

The above-mentioned approaches use very different concepts, but they all share a common signature: they start from a selected protein or metabolite as bait. These methods allow the exploration of interactions of a target protein or small molecule, but they cannot provide a panoramic picture of the whole interactome.

The non-targeted PROMIS (PROtein-Metabolite Interactions using Size separation) strategy allows PMI analysis under native conditions. Protein–metabolite complexes are separated by size exclusion chromatography, followed by quantitative metabolomic and proteomic analyses of the resulting fractions. The definition of putative intermolecular interactions is based on coelution [49].

The main advantages of PROMIS are its ability to work at in vivo concentrations and the absence of the need to modify small molecules or proteins. However, by its very nature, coelution is a sign but not a proof of interaction. PROMIS should be considered an exploratory approach for constructing an interactome map, which should be combined with orthogonal methods to refine the composition of complexes. Like gene expression studies, integrating multiple PROMIS datasets will likely be sufficient to double-check putative interaction [71]. Recently, the application of PROMIS to *Saccharomyces cerevisiae* cells allowed for the detection of interaction events between 3982 proteins and 74 small molecules, and most of these interactions were detected for the first time [70].

The ideologically similar TICC (target identification by chromatographic coelution) method is based on identifying a characteristic shift in the chromatographic elution profile of a compound bound to a protein target. TICC was used to test known drug–protein interactions (micromolar to nanomolar Kd) in a native cell lysate or lysate doped with the molecules of interest. The idea is that the recombinant protein is incubated with a mixture of compounds; unbound small molecules are separated via single-stage size exclusion chromatography, and interacting compounds are determined using metabolomics methods. In addition to size exclusion, ion exchange chromatography has also shown the ability to separate free compounds from those bound with proteins.

### 3.4. Biophysical Approaches

The methods mentioned earlier allow the detection of PMIs in panoramic mode but do not provide information about these interactions’ kinetics, degree of affinity, and specificity. For these purposes, various biophysical methods (such as surface plasmon resonance (SPR), isothermal titration calorimetry (ITC), and nuclear magnetic resonance (NMR) spectroscopy) are used that specialize in the rigorous determination of interaction properties [72].

In the Surface Plasmon Resonance (SPR) method, a protein of interest is immobilized on a sensor chip, and upon binding of a metabolite, the chip detects the change in the refractive index of the complex [73]. SPR requires small (in submicromolar range) amounts of analytes and allows measurement of kinetic parameters, as well as the dissociation constant (Kd). Consequently, this method is widely used to define the parameters of interaction between particular pairs of proteins and metabolites [74,75,76]. As an example, in a recent investigation dedicated to studying the interactions between an enzyme CYP51A1 and flavonoids, this enzyme was shown to be interacting with baicalein, luteolin and luteolin 7,3′-disulfate and the dissociation constants were defined in each case [76]. This method has a limitation in that metabolites with a smaller molecular mass induce only poorly detectable changes in the refractive index, but the sensitivity of the method can be improved significantly with gold nanoparticle chips [77].

Isothermal Titration Calorimetry (ITC) is a solution-based and label-free direct quantitative method measuring the change in heat that is released or absorbed upon interaction between two molecules. This allows us to directly measure such fundamental thermodynamic parameters of binding as the binding affinity or the dissociation constant. As an example of its application, it was utilized in a study of interaction parameters between lysozyme and its inhibitors, *N*-acetyl glucosamine trimer (NAG_3_) and monomer NAG [78]. This method is powerful enough to reveal the specifics of the nature of the interaction, such as the influence of the hydrophobic effect [79] or the exact contributions of solvation and protonation to the binding [80]. In some experimental setups, it is possible to measure the thermal capacity and conformational equilibrium of the binding [81]. Although ITC does not require any surface immobilization of the protein or its modification, it requires relatively large amounts of analytes, which may be problematic in the analysis of proteins available in small quantities only.

Nuclear Magnetic Resonance (NMR) spectroscopy is a technique in which radio frequency waves are used to induce changes in the energy levels of certain atomic nuclei subjected to a strong magnetic field. In the context of PMI studies, NMR is the method of choice for obtaining structural information about the protein–metabolite complex [82,83]. The approach is so capacious that it is possible to construct a classification similar to the structure of this paper: from metabolite to protein [84] from protein to metabolite [85]. Advantages of NMR include versatility, sensitivity and upcoming potential to conduct in vivo studies [86]. A shortcoming of the base method is its limited capacity for working with large (≥50 KDa) proteins, but this limitation can be circumvented by the use of several modifications of the method, such as the Nuclear Overhauser Effect (NOE) or Saturation-Transfer Difference (STD) [83]. In addition to the methods described above, there are several techniques based on fluorescence spectroscopy, Fourier Transform Infrared (FTIR) and Raman spectroscopy, analytical ultracentrifugation (AUC), and many other approaches [72,87,88], which allow the measurement of the influence of conditions (pH, ion concentrations, temperature) on the interaction of a particular protein–metabolite pair while also allowing the detection of conformational changes in protein molecules upon binding with the metabolite.

Cryogenic electron microscopy (Cryo-EM) is one of the key methods for resolving the macromolecular structures of proteins, including their complexes with substrates or even intermediate states [89]. Samples for Cryo-EM are rapidly cooled to cryogenic temperatures, which prevents the crystallization of water molecules and preserves the native sample structure. Thus, in contrast to, for example, X-ray crystallography, Cryo-EM does not require a resource-intensive and technically challenging step in protein crystallization. Subsequently, frozen samples are processed and analyzed with a transmission electron microscope (TEM). In the realm of protein–metabolite interactomics, Cryo-EM has found its use in the investigation of the structures of protein–ligand complexes. For example, it was utilized to resolve structures of glutamate dehydrogenase (GDH) interacting with GTP and/or NADH, revealing how these small molecules mechanistically regulate the activity of GDH [90]. Therefore, Cryo-EM is an important tool that can be used to confirm and annotate PMIs discovered in high-throughput studies.

The microscale thermophoresis (MST) technique utilizes the directed movements of macromolecules in the temperature gradient to detect interactions, including those between proteins and metabolites. In brief, the concentration of macromolecules, identified via fluorescence, decreases in the heated area since macromolecules move to colder zones. Binding of a ligand to a protein can induce changes in the size, charge, hydration shell or conformation of the resulting complex. This can affect the movement of a protein in the temperature gradient, which permits quantification of the interaction constant between the protein and the metabolite [91]. This approach was recently applied to study the interaction between RhoA and Rhotekin-BD [92]. Microscale thermophoresis is relatively easy-to-use and can detect interactions in a wide range of affinities (mM to pM). The main drawback of this method is the requirement for fluorescent samples or probes.

Each biophysical method has its limitations and application areas; thus, a comprehensive understanding of thermodynamic, kinetic, structural and dynamic properties of a particular interaction is achieved by a combination of the methods. For instance, FTIR and Raman spectroscopy complement each other quite well: the former is not recommended for water-containing samples, while the latter is the method of choice for such samples. On the other hand, the sensitivity of Raman spectroscopy is drastically reduced in colored or fluorescent samples, but this is not a problem for FTIR spectroscopy. Since the vast majority of biophysical methods are carried out in vitro (except for NMR analysis adapted for native conditions), the results obtained in such studies cannot be unequivocally transferred to a “cellular” environment. For a correct interpretation of such data, additional experiments should be carried out with methods that work in conditions close to native ones. A brief description of the principles of the methods reviewed in this manuscript, as well as their strengths and weaknesses, are listed in Table 1.

## 4. Databases

The data itself is often not enough, even in the field of the omics sciences, in which it is common to initiate research projects based on not hypotheses but rather experimental data. New knowledge emerges from the intersection of diverse data in a new way that facilitates data exploration for powerful and systematic analysis. Genomics has passed this path, and now proteomics and metabolomics are passing it, too [95]. Public databases for storage, organization and comparative analysis of gathered data are vitally important to support this advancement. Interactomics (and in particular the field of PMIs) is no exception and cannot be considered a truly mature omics-based science without having established databases that provide convenient access to all the related data.

Although the field of protein–metabolite interactomics is still relatively young, there are already a number of databases that contain quantitative and qualitative information regarding the results of experiments studying protein–metabolite interactions. Experimental confirmation of PMIs is a complicated task, so the amount of knowledge in this area lags behind PPIs. To date, there are 17 databases that aggregate information about PMIs, but fewer resources are regularly updated and curated. Several key databases are listed in Table 2.

From this list of the most influential PMI-related databases, it is apparent that most of the databases are devoted to clinically relevant compounds and some of the databases are protein-centric, rather than metabolite-centric. Thus far, only one of the listed databases, PMI-DB, significantly leverages the aforementioned new high-throughput techniques for capturing PMIs. This reflects the very young state of research into PMIs from a panoramic point of view. However, most of the presented databases are still indispensable for such investigations, as they provide high-quality, ground truth information that is used to annotate PMIs captured during the experiment and separate well-known interactions from new ones. In the near future, we expect that the quantity and quality of PMI-centric databases will grow significantly, reflecting the fast rate of development and application of new technologies for the detection of PMIs.

## 5. Conclusions

Interactomics, including protein–metabolite interactomics, sits squarely at the intersection of chemistry, biology and bioinformatics, providing an opportunity for systematic studies of the transfer of biological information from one layer to another. A close relationship between the proteome and the metabolome has been demonstrated by the recent development of several models for predicting changes in the metabolome from the proteome [111,112]. Such approaches commonly utilize genome-scale metabolic models that have been developed primarily based on literature curation [113]. However, even the best current genome-scale metabolic models are considered incomplete, thus limiting their ability to predict metabolic changes from proteomic/transcriptomic data [114]. Large-scale investigations of PMIs can thus provide missing information and help build more powerful predictive models.

Accordingly, in a larger context, knowledge of protein–metabolite interactions is vital for insightful interpretation of multi-omics experiments. In such experiments, several layers of omics data from the same samples are combined to provide the most information possible about the experimental system, for instance, a model of disease [115]. Typically, these omics layers include transcriptome, proteome and metabolome, although other layers are being increasingly leveraged to supplement the analysis [116]. For such analyses, the choice of a strategy for combining information from different omics layers is critical. In the simplest form, the integration can be performed by independent pathway enrichment for each omics type and a subsequent combination of statistical results for each detected pathway [117]. Other, more nuanced methods attempt to first integrate all detected biological entities into networks and then perform the analysis using, for example, graph-based approaches [118]. However, all of these methods depend heavily on pathway topology data, which are typically provided by databases such as KEGG [119] and Reactome [120]. Consequently, in order to most fully integrate metabolomic data with other omics layers, it is important to have a complete picture of protein–metabolite interactions in a given experimental model.

While the importance of detecting and describing protein–metabolite interactions is undoubtedly significant, this area of research is still quite early in its development and has several opportunities for much-needed improvements, especially in the context of high-throughput experiments. The first of these is the need for a systematic and standardized procedure of reporting and integrating experiments. As demonstrated in the present review, there are numerous methods for detecting protein–metabolite interactions, which have widely different requirements and capabilities. Efforts to systematically compare and integrate data from multiple methods, such as the one attempted by the PMI-DB database, are going to be critical for the confident use of data from interactomics experiments, as has been demonstrated for a very similar but more mature field of protein–protein interactomics [1]. Another problem, which is more fundamental for the field of interactomics and biology in general, is the fact that the majority of high-throughput methods detect the fact of physical association between interacting molecules but lack ways to describe the nature of association, i.e., biological role [121]. While some of the detected associations can be further characterized in vitro and in vivo, pointing to their function [35], such methods are significantly less high-throughput, which makes the annotation of all detected interactions extremely challenging. Accordingly, the number of detected interactions from all experiments significantly outpaces the volume of information about the nature of the interactions, thus limiting their utility in future investigations. Considering the weak and transient nature of many reported interactions, as well as the relatively high rate of false-positive results typical of interactomics techniques [122], validation and subsequent annotation of the detected interactions must be accelerated to make the most of the wealth of data from interactomics experiments.

In spite of all the challenges, the field of protein–metabolite interactomics enters an exciting phase, where freshly developed high-throughput technologies can be applied to many objects of interest, revealing an underappreciated layer of biological complexity. Such information about unannotated interactions between proteins and metabolites can be further annotated by ever-improving targeted structural and functional assays. Overall, considering the importance of the proteome and the metabolome layers in biological systems, as well as the intensive cross-talk between them, the field of protein–metabolite interactomics has the potential to solve many riveting questions in health and disease.

## Figures and Tables

**Table 1:** Summary of the principles of the methods described in the text, as well as their strengths and weaknesses.

Method	Concept	Advantages	Limitations	References
Small molecule to protein				
AP	Protein affinity for immobilized small molecule ligand	Proteome-wide scale;	Requires modification of small molecules. High false-positive rate	[21,24,26,27]
LiP-MS	Changes in the stability of the protein–metabolite complex to proteases	Proteome-wide scale; Does not require modification of small molecules; Identification of the ligand-binding site	False-negative results Competition with endogenous metabolites	[28,29]
SPROX	Changes in the stability of the protein–metabolite complex to oxidation	Proteome-wide scale; Does not require modification of small molecules;	False-negative results Competition with endogenous metabolites	[36,37,39]
CETSA/TPP	Changes in the stability of the protein–metabolite complex to thermal effects	Proteome-wide scale; Does not require modification of small molecules;	False-negative results Competition with endogenous metabolites	[42,43,44]
PISA	Changes in the stability of the protein–metabolite complex to thermal effects	Proteome-wide scale; Does not require modification of small molecules; Increased throughput compared to CESTA/TPP	False-negative results Competition with endogenous metabolites	[50,51,52]
SIP	Changes in the stability of the protein–metabolite complex to solvent precipitation	Proteome-wide scale; Does not require modification of small molecules;	False-negative results Competition with endogenous metabolites	[53,54,55]
Chemoproteomic profiling	Chemical functionalization of a small molecule leading to covalent binding to a protein target	Proteome-wide scale; Captures weak binding	Requires modification of small molecules; Time-consuming	[56,59,60]
Protein to small molecule				
NMR	Investigation of protein–metabolite complex structure using nuclear magnetic resonance	Allows obtaining structural information about the protein–metabolite complex Sensitivity	Limited possibility of analyzing proteins heavier than 50 KDa In vitro analysis	[49,63,66]
MIDAS	Investigation of protein–metabolite complex structure using nuclear magnetic resonance	Identification of both high-affinity and low-affinity interactions	Requires large amount of purified proteins In vitro analysis	[65]
DRaCALA	Immobility of protein–bound metabolite on nitrocellulose membrane	Simplicity of implementation	The need to use isotope-labeled standards In vitro analysis	[64,67,68]
TAP	Purification of an epitope-labeled protein in complex with a small molecule	Metabolome-wide scale; Finds both protein small molecule partners;	High rate of false positive results. Requires protein labeling	[69,70]
Non-targeted				
PROMIS	Coelution of protein and metabolite forming complex	On the scale of the proteome and metabolome; Does not require modification of small molecules or protein labeling Possibility of simultaneous detection with protein–protein interactions	Coelution is an indication, but not proof of interaction	[70,71]
Biophysical approaches				
SPR	Change in refractive index when a protein interacts with a metabolite	Allows to measure: kinetic parameters, dissociation constant (Kd)	Reduced sensitivity to very low molecular weight metabolites. Requires protein immobilization In vitro assay	[73,74,75]
ITC	Measurement of heat released or absorbed during the interaction of a protein and a metabolite	Allows to measure: kinetic parameters, dissociation constant (Kd) Allows evaluation of the nature of the interaction	Requires large amounts of purified proteins and metabolites In vitro assay	[78,79,93]
NMR	Investigation of protein–metabolite complex structure using nuclear magnetic resonance	Allows obtaining structural information about the protein–metabolite complex Sensitivity	Limited possibility of analyzing proteins heavier than 50 KDa In vitro analysis	[82,83,84,85,86]
Cryogenic electron microscopy	Investigation of protein–metabolite complex structure using electron cryo-microscopy	Allows obtaining structural information about the protein–metabolite complex Sensitivity Easier sample preparation	Limited databases of small molecules	[89,90,94]
Microscale thermophoresis	Change of mobility in the interaction of a protein with a metabolite	Allows to measure dissociation constant in wide range (Kd) Fast measurement Measurements of complex mixtures	Requirement for fluorescent samples	[91,92]

**Table 2:** Databases with information on PMIs.

Database	Release Date	Type of Data	Distinctive Features	Last Update Date	References
Protein Data Bank (PDB)	1971	Protein structures obtained using X-ray, NMR and Cryo-EM	Structures for known complexes of proteins with small molecules available via LigandExpo tool	Each week	[96,97,98]
BRaunschweig ENzyme DAtabase (BRENDA)	1987	Literature-curated reconstruction of metabolic pathways	Data for approx. 90,000 enzymes from approx. 13,000 organisms and more than 207,000 small molecules	July 2022	[99,100]
ChEMBL	2009	Detailed information for small molecule compounds	Data for approx. 2.3 million compounds Information on how small molecules interact with protein targets	July 2022	[101,102,103]
DrugBank	2006	Detailed information for clinically relevant small molecules	Data for FDA-approved (or in the process of being approved) drugs MS and NMR spectra available Information for the protein targets of many drugs	January 2023	[104,105,106]
BindingDB	2001	Quantitative information for interactions between proteins and drug-like molecules (IC50, Ki or Kd)	Data for approx. 2.6 million binding events between 8946 proteins and 1,129,664 drug-like molecules Data for computationally docked conformations	December 2022	[107,108]
Therapeutic Target Database (TTD)	2002	Literature-curated information for interactions between proteins and small molecule drugs including weak or even non-binders	Contains data for 38,760 drugs and 3578 targets Definition of strong interactions: IC50 < 50 µM; weak: 50 µM < IC50 < 200 µM; rest are non-interactions	September 2021	[109]
PMI-DB	2021	A collection of literature-derived 49,785 interaction events between 9631 proteins and 23 small molecules including non-interactions	Data from studies using six different techniques (LiP-SMap, CETSA/TPP, SPROX, flavonoid, lipid and sterol probes) Machine learning models for predicting small molecule binding profiles	Twice annually	[110]

Abbreviations: NMR—Nuclear magnetic resonance, Cryo-EM—Cryogenic electron microscopy, FDA—U.S. Food and Drug Administration.

## Data Availability

Not applicable.
